# Supplementary material for: The Relationship between Gut Microbiome and Cognition in Older Australians
Source: Nutrients. 2021 Dec 24;14(1):64. doi: 10.3390/nu14010064 (PMC8746300; doi:10.3390/nu14010064)
Supplement: Supplementary file 1 [file nutrients-14-00064-s001.zip › nutrients-1486573-supplementary.pdf]

Supplementary Materials:

Table S1: CDR factors scoring

CDR factor	Scoring
Quality of episodic secondary memory (QESM)	(Word recall original accuracy + Word recall novel accuracy- 100) + (Picture recall original accuracy + Picture recall novel accuracy -100) +((Immediate word recall accuracy * 15/100 -Immediate word recall error) * 100/15) + ((Delayed word recall accuracy * 15/100 – Delayed word recall error *100/15)
Quality of working memory (QWM)	Spatial working memory sensitivity index + Numeric working memory sensitivity index
Power of Concentration (PoC)	Simple reaction time + Digit vigilance + Choice reaction time
Speed of memory (SoM)	Spatial working memory reaction time + Numeric working memory reaction time + Word recall reaction time + Picture recall reaction time
Continuity of attention (CoA)	(Digit vigilance accuracy *45/100) + (Choice reaction time accuracy *50/100) – Digit vigilance false alarms

QESM, Quality of episodic secondary memory; QWM, Quality of working memory; PoC, Power of concentration; CoA, Continuity of attention; SoM, Speed of memory

Table S2: Prevalence of gut microbiome identified in the cohort.

Prevalence	Total Abundance	Family
33	96	Actinomycetaceae
10	14	Micrococcaceae
13	59	Propionibacteriaceae
11	19	Bifidobacteriaceae
69	6869	Coriobacteriaceae
69	996750	Bacteroidaceae
68	30648	Barnesiellaceae
65	11556	Odoribacteraceae
61	26455	Paraprevotellaceae
64	6163	Porphyromonadaceae
68	308537	Prevotellaceae
69	183261	Rikenellaceae
61	29806	S24-7
6	42	Elusimicrobiaceae
6	7	Listeriaceae
18	22	Gemellaceae
6	10	Aerococcaceae
23	60	Carnobacteriaceae
31	246	Lactobacillaceae
2	2	Leuconostocaceae

68	2105	Streptococcaceae
31	260	Turicibacteraceae
66	30470	Christensenellaceae
69	30888	Clostridiaceae
29	207	Dehalobacteriaceae
10	65	EtOH8
69	401499	Lachnospiraceae
65	4984	Mogibacteriaceae
45	392	Peptococcaceae
5	33	Peptostreptococcaceae
69	693321	Ruminococcaceae
26	208	Tissierellaceae
69	105751	Veillonellaceae
69	50024	Erysipelotrichaceae
28	31746	Fusobacteriaceae
43	7270	Victivallaceae
8	19	Rhodobacteraceae
69	40178	Alcaligenaceae
3	8	Burkholderiaceae
2	3	Comamonadaceae
43	753	Oxalobacteraceae
8	39	Neisseriaceae
66	10264	Desulfovibrionaceae
11	31	Campylobacteraceae
18	56	Helicobacteraceae
1	51	Succinivibrionaceae
67	40618	Enterobacteriaceae
32	1442	Pasteurellaceae
1	9	Pseudomonadaceae
29	1007	Synergistaceae
17	5171	Anaeroplasmataceae
9	15	Deinococcaceae
11	24	Rs-045
34	1717	Cerasicoccaceae
67	185476	Verrucomicrobiaceae
69	447944	Unknown
